# Perioperative pain-anxiety response profiles and their predictors in patients undergoing dental implant surgery: a latent profile analysis

**DOI:** 10.3389/fpubh.2026.1885978

**Published:** 2026-07-27

**Authors:** Yuqing Zhang, Shumin Nie, Xiaoli Ren

**Affiliations:** 1 Shandong Provincial Hospital Affiliated to Shandong First Medical University, Jinan, Shandong, China; 2 School of Nursing, Shandong First Medical University, Tai'an, Shandong, China

**Keywords:** anxiety, dental implant, latent profile analysis, pain, predictive factors

## Abstract

**Background:**

Pain and anxiety are common perioperative experiences among patients undergoing dental implant surgery; however, their dynamic interaction patterns and interindividual heterogeneity remain poorly characterized.

**Aim:**

This study aimed to characterize perioperative pain–anxiety dynamics in dental implant patients, identify latent response profiles, and examine predictors to support early risk stratification and personalized nursing care.

**Methods:**

A prospective observational study with repeated perioperative assessments was conducted in 204 dental implant outpatients. Predictors included trait-like dental anxiety (MDAS score), surgical factors (bone graft use and number of implants), smoking status, and family accompaniment. Perioperative pain intensity and state anxiety were measured using the Visual Analog Scale (VAS) and the Visual Analog Scale for Anxiety (VAS-A), respectively, at five perioperative time points. Repeated-measure latent profile analysis identified pain-anxiety response profiles, and binary logistic regression examined predictors of profile membership.

**Results:**

Two distinct profiles were identified: a hypo-reactive profile (64.2%) and a hyper-reactive profile (35.8%). Hyper-reactive patients showed higher preoperative anxiety and postoperative pain, peaking intraoperatively and at 6 hours postoperatively. Higher MDAS scores, greater surgical complexity, and absence of family accompaniment significantly predicted hyper-reactivity, whereas smoking was associated with a lower likelihood of hyper-reactivity.

**Conclusion:**

Perioperative pain and anxiety exhibit substantial interindividual variability among dental implant patients, with two distinct response profiles identified: hypo-reactive profile and hyper-reactive profile. Preoperative anxiety, surgical complexity, and social support significantly influence response types. Early identification of high-risk response profiles may facilitate stratified, patient-centered nursing interventions to optimize perioperative management and recovery outcomes.

## 1 Introduction

Oral diseases constitute one of the most significant global health burdens, affecting more than 3.6 billion individuals worldwide, with edentulism alone accounting for over 18 million cases (1). Dental implant rehabilitation, which provides patients with missing teeth or edentulism with restorations that closely resemble natural dentition in appearance and offer substantial masticatory function, has therefore gained widespread acceptance as a predictable and reliable therapeutic option (2). Over recent decades, the use of dental implants has increased markedly, largely attributable to their high success rates and favorable health-related outcomes (3, 4). Beyond restoring masticatory efficiency and supporting adequate nutritional intake, implant therapy may confer additional systemic benefits, including a potential reduction in cognitive decline and cardiovascular disease risk (1, 5). Moreover, the high esthetic similarity between implants and natural teeth has been shown to enhance patients' perceived appearance and psychological well-being, thereby improving social confidence and overall life satisfaction (6). Despite these well-documented advantages, implant-based rehabilitation is accompanied by distinct perioperative challenges that warrant further attention.

As an inherently invasive therapeutic procedure, dental implant surgery is frequently accompanied by varying degrees of preoperative tension, anxiety, and perioperative pain experiences. Dental anxiety is one of the most common anxiety-related conditions in dental practice, and is characterized by an irrational and disproportionate negative emotion of dental procedures, typically accompanied by pronounced physiological and emotional arousal (7). Previous studies have reported that the prevalence of dental anxiety among patients with oral diseases ranges from 62.0% to 79.2% (8), with manifestations spanning from mild discomfort to severe phobic reactions, exerting both psychological and physiological consequences on affected individuals (9). Psychological vulnerability and negative emotional states play a critical role in shaping pain perception and subjective pain intensity. Evidence suggests that patients with higher levels of anxiety tend to report greater pain intensity during dental treatment compared with those experiencing lower anxiety levels, which may subsequently compromise treatment adherence or lead to treatment avoidance (10, 11). Such avoidance behaviors can result in delayed intervention, missed optimal treatment windows, and further deterioration of oral health conditions (12).

Previous research has commonly conceptualized anxiety along two dimensions: trait anxiety, which reflects a relatively stable psychological predisposition, and state anxiety, which represents a transient psychological response elicited by specific situational contexts (13). Within dental care settings, anxiety has been widely recognized as a key psychological factor influencing patients' pain experiences (14). Existing evidence suggests that, among patients with dentition defects undergoing implant treatment, higher levels of preoperative trait anxiety are significantly associated with greater postoperative pain perception (15). A longitudinal study has further indicated that (16), although postoperative pain following implant surgery generally demonstrates a gradual decline over time, the magnitude of preoperative anxiety may shape differential pain perceptions across distinct postoperative time points. Concurrently, a recent study has demonstrated that dental anxiety can exert a sustained influence on pain perception throughout the entire course of dental treatment, thereby exacerbating patients' adverse treatment experiences (16).

However, evidence regarding the relationship between dental anxiety and pain among patients undergoing dental implant treatment remains inconclusive. Some studies have reported that only severe levels of preoperative dental anxiety are significantly associated with intraoperative pain perception, whereas mild to moderate anxiety appears to exert no substantial effect (17). Other investigations have failed to identify a significant association between dental anxiety and postoperative pain (18). One possible explanation for these inconsistent findings is that previous studies have largely treated dental implant patients as a homogeneous population, overlooking potential heterogeneity in perioperative psychological and pain responses. Moreover, pain and anxiety are inherently dynamic experiences that fluctuate throughout the perioperative period.

Nevertheless, most existing studies have relied on cross-sectional designs or assessed anxiety and pain at only a limited number of perioperative time points (19–21), limiting the ability to capture dynamic fluctuations in state anxiety throughout the perioperative period. Consequently, the temporal evolution of anxiety–pain responses remains insufficiently understood. In addition, considerable disagreement persists regarding the timing of peak postoperative pain following dental implant surgery (22–24), suggesting that single–time-point assessments may be inadequate for capturing the full trajectory of patients' perioperative experiences. Furthermore, conventional variable-centered analytical approaches primarily focus on average population estimates and may obscure clinically meaningful differences between individuals. As a result, distinct anxiety–pain response patterns that potentially exist among dental implant patients remain largely unexplored. To date, it remains unclear whether patients undergoing dental implant therapy can be classified into distinct perioperative anxiety–pain response profiles and which factors predispose individuals to higher-risk response patterns.

Against this background, there is a clear need to adopt a person-centered perspective and integrate multi-time-point assessments to achieve a more refined characterization of the dynamic interplay between perioperative anxiety and pain among patients undergoing dental implant treatment. Latent profile analysis (LPA), a pattern-oriented analytic approach, enables the identification of latent subgroups characterized by distinct anxiety–pain response profiles within patient populations, thereby elucidating heterogeneity in psychological and physiological responses across individuals. By distinguishing subgroups with varying levels of response risk, this approach may assist nurses in implementing targeted psychological interventions and pain management strategies at an early perioperative stage, optimizing clinical decision-making and enhancing patients' adherence to dental treatment as well as their overall treatment experience.

Accordingly, this study aimed to explore the dynamic characteristics of perioperative anxiety and pain among patients undergoing dental implant therapy, to identify latent anxiety–pain response profiles, and to further examine associated influencing factors. By supporting risk stratification and individualized decision-making in perioperative nursing care, the findings are expected to provide evidence for the development of time-specific and precision-oriented anxiety alleviation and pain management strategies, ultimately improving the safety of implant treatment, patient experience, and overall quality of nursing care.

## 2 Materials and methods

### 2.1 Study design/sample

**Figure 1:**
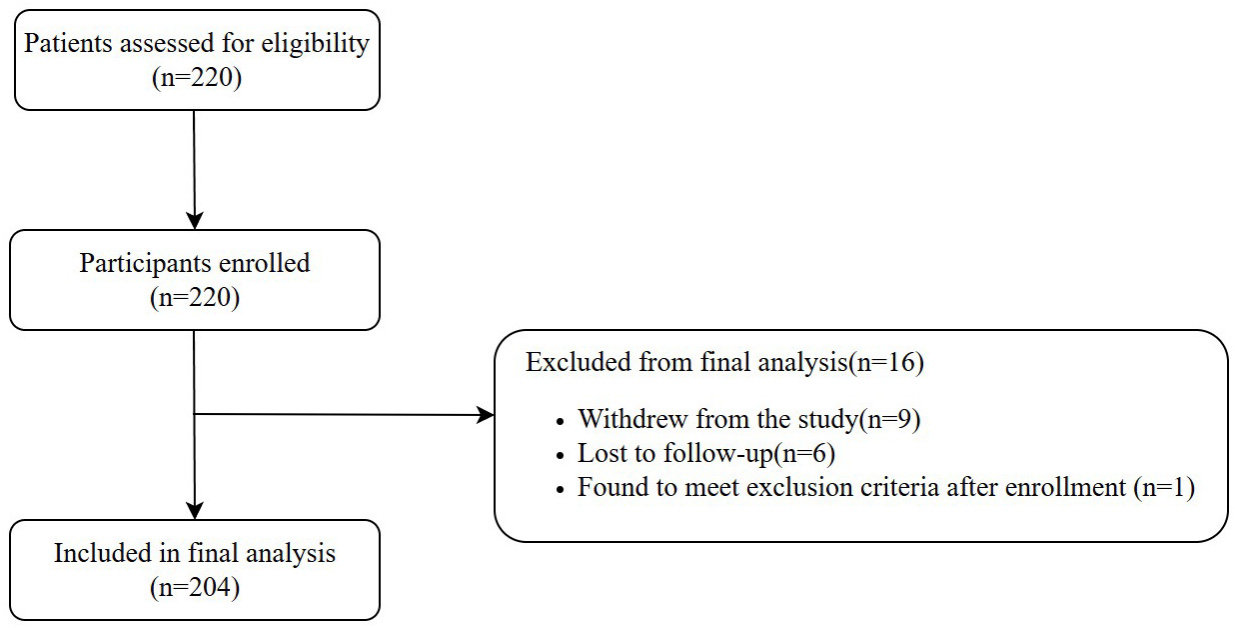
Flow diagram of participant recruitment and study retention. A total of 220 patients scheduled for dental implant surgery were enrolled and completed baseline assessment. 16 participants were excluded from the final analysis because of voluntary withdrawal from the study (*n* = 9), loss to follow-up (*n* = 6), or found to meet exclusion criteria after enrollment (*n* = 1). The final analytical sample consisted of 204 participants who were included in the repeated-measure latent profile analysis and subsequent binary logistic regression analysis.

Convenience sampling was used because of the exploratory nature of the study and the need for repeated perioperative follow-up assessments. Patients who received dental implant surgery in the Department of Stomatology at Shandong Provincial Hospital Affiliated to Shandong First Medical University from November 2023 to February 2024 were selected as the subjects. The inclusion criteria were patients aged ≥ 18 years, with no restrictions on sex; patients undergoing single- or multiple-tooth dental implant rehabilitation; patients with adequate communication and comprehension abilities to complete follow-up assessments and study questionnaires; and patients who provided written informed consent and voluntarily agreed to participate in the study. The exclusion criteria were as follows: a history of psychiatric disorders or use of antianxiety or analgesic medications within the previous year; systemic diseases, psychological disorders, or physical illnesses that might influence pain thresholds; cognitive impairment or visual/hearing dysfunction that could interfere with completion of repeated assessments; and medical contraindications for dental implant surgery. The participant recruitment and study retention process is illustrated in Figure 1.

### 2.2 Variables

#### 2.2.1 Predictor variables

The primary predictor variables in this study included preoperative dental anxiety, surgical-related factors, social support, and smoking status. Smoking status was included because previous evidence suggests that smoking may influence pain perception and postoperative recovery outcomes, and was therefore considered a potentially relevant predictor of perioperative anxiety–pain response profiles (25, 26). Because the Modified Dental Anxiety Scale (MDAS) measures trait-like dental anxiety, it was treated as a baseline predictor and was not included among the repeated anxiety indicators used in the latent profile analysis. Surgical-related factors included the use of bone graft material, the number of implants placed, and the type of implant procedure. Social support was operationalized as the presence or absence of family members or caregivers accompanying the patient during treatment. Smoking status was recorded as a binary variable (yes/no).

#### 2.2.2 Outcome variables

The primary outcome variable was perioperative anxiety–pain response profile membership, identified through repeated-measure latent profile analysis (R-LPA). Anxiety and pain were repeatedly assessed at five perioperative time points: preoperative, intraoperative, 6 h postoperatively, 24 h postoperatively, and 7 days postoperatively. The T1 assessment was performed immediately after surgery. Patients retrospectively evaluated the level of anxiety and pain they experienced during the implant procedure using the VAS-A and VAS-P. State anxiety was measured using the Visual Analog Scale for Anxiety (VAS-A), and pain intensity was measured using the Visual Analog Scale for Pain (VAS). Based on the joint trajectories of anxiety and pain across time, patients were classified into two distinct profiles: a hypo-reactive profile and a hyper-reactive profile.

#### 2.2.3 Other variables

In addition to the primary predictors and outcomes, demographic characteristics including age, sex, oral health and educational level were collected for descriptive purposes only and were not included as covariates in the inferential analyses.

### 2.3 Data collection methods

#### 2.3.1 General information questionnaire

The questionnaire was compiled by the researcher combined with the actual dental clinical practice, including: gender, age, education level, occupational category, level of pain tolerance, the number of dental implants, accompanied by family members or not, and used bone powder or not.

#### 2.3.2 Modified dental anxiety scale (MDAS)

The modified version of the MDAS verified and establish by Qian et al. (27, 28) was used to investigate the patients' dental anxiety. The scale contains 5 entries involving different moments that may lead to an increase in anxiety during dental treatment. Both reliability and validity are good. This scale is a classic tool for assessing pre-dental treatment classic tool for trait anxiety (20). MDAS is based on a 5-point Likert scale. Each entry rated from 1 to 5 (most calm to most anxious) and a total score of 5 to 25, higher scores indicating higher levels of dental anxiety, when the MDAS score value of ≥12 is considered to have significant dental anxiety (27, 28).

#### 2.3.3 Visual analog scale (VAS)

In this study, the VAS was used to assess patients' pain perception and anxiety levels during the perioperative period (29, 30). The assessment was done using a standard 10 cm straight line with a score of 0 to 10 (not at all ~ extreme). The Visual Analog Scale-Anxiety (VAS-A) has been widely validated as an easy and sensitive anxiety assessment tool, especially for state anxiety monitoring in the perioperative period (31, 32).

#### 2.3.4 The 14-item Oral Health impact profile (OHIP-14)

In this study, oral health-related quality of life (OHRQoL) was assessed using the 14-item Oral Health Impact Profile (OHIP-14). The OHIP-14 was originally developed by Slade and Spencer and was subsequently translated and validated in Chinese populations. The instrument comprises 14 items covering four domains: reduced independence, psychological discomfort, physical functional discomfort, and pain and discomfort. Responses are rated on a 5-point Likert scale ranging from 0 (“never”) to 4 (“very often”), resulting in a total score between 0 and 56. Higher scores indicate poorer OHRQoL and a greater negative impact of oral conditions on daily functioning and well-being (33).

#### 2.3.5 Data collection and quality control

All surveys used a combination of paper questionnaires and timed multiple time-point assessments, and were guided by uniformly trained research assistants in the waiting and treatment areas of the outpatient clinic. Oral dental implants are made by implanting an artificially manufactured tooth root into the alveolar bone at the site of the missing tooth, and after it has bonded with the alveolar bone, the artificial root is utilized to support a crown that is similar in shape to the tooth. Compared to other restorative methods, dental implants can perform chewing functions independently without relying on adjacent natural teeth. Data were obtained in the perioperative period using a combination of on-site completion or telephone callbacks. Each subject completed the pain score and anxiety assessment before (T0), during (T1), 6h (T2), 24h (T3), and 7d (T4) after operation to ensure the temporal consistency and longitudinal integrity of the data. Investigators on-site verified the completeness of the data and uniformly entered into Excel, and double data entry with verification ensured the accuracy. A total of 220 questionnaires were distributed in this study, and 204 valid questionnaires were recovered, with a valid recovery rate of 92.73%.

### 2.4 Data analyses

Mplus 8.3 software was used for LPA modeling (34) and SPSS 27.0 was used for descriptive statistics and regression analysis. Starting from an initial model with a single category, the optimal model was determined by gradually increasing the number of categories. The best model was determined using the aikek information guidelines (AIC), bayesian information criterion (BIC), sample-corrected Bayesian information criterion (aBIC), entropy, romondaleu Entropy, LoMendell Rubin adjusted likelihood ratio (LMR) and Bootstrap likelihood ratio test (BLRT) were used as model evaluation metrics, AIC, BIC and aBIC were used to assess the AIC, BIC and aBIC were used to evaluate the model fit, the smaller the value, the better the fit; Entropy indicator was used to evaluate the classification accuracy, the closer the value was to 1, the better the fit; LMR and BLRT could indicate the model fitness. SPSS27.0 software was used to data analysis. Measurement data conforming to normal distribution were described by mean ± standard deviation; count data were expressed by frequency and constitutive ratio. Variables demonstrating potential associations in univariate analyses, together with factors considered clinically relevant based on previous literature, were included in the binary logistic regression model. Prior to model construction, multicollinearity among independent variables was assessed using tolerance statistics and variance inflation factors (VIFs). Model fit was evaluated using the Hosmer–Lemeshow goodness-of-fit test. Statistical significance was defined as a two-sided *P* value <0.05.

### 2.5 Ethical considerations

The Ethics Committee of Shandong Provincial Hospital Affiliated to Shandong First Medical University approved this study (SWYX: NO. 2023-1044), and the study was conducted in accordance with the Declaration of Helsinki (2013). The purpose and significance of this study were explained in detail to the study participants before data collection, and the study participants gave informed consent.

## 3 Results

### 3.1 Results of latent profile analysis of perioperative anxiety and pain in dental implant outpatients

In this study, the VSA scores of perioperative anxiety and pain at 5 time points were used as the observables, and the LPA method was used to identify the trajectory of the patient's psychophysiological responses in the perioperative recovery period. Four categories of models were established by comparison of model fitting. The results showed (Table 1), which shows that as the number of type increased, the AIC, BIC, and aBIC indicators gradually decreased, and the entropy values of the models were all >0.90, indicating higher classification clarity. However, in the Lo–Mendell–Rubin test (LMRT), the 2-type model was significantly compared with the 1-type model (*P* = 0.023), while the difference between 3-type model and 2-type model was not significant (*P* = 0.513). Combining the model interpretability and clinical interpretability, the 2-type model was finally selected as the best fit. The first category (*n* = 131, 64%) showed low levels of anxiety and pain throughout the perioperative period and was named “Hypo-reactive”, The second category (*n* = 73, 36%) showed significantly higher preoperative anxiety than the first category and was named “Hyper-reactive”, it has a significant postoperative pain response.

**Table 1:** Potential Profile Model Fitting indicators for perioperative anxiety and pain in dental implant patients.

Model	Log-likelihood	AIC	BIC	aBIC	LMRT *P*-value	BLRT *P*-value	Entropy	Category probability
1-type	−3,641.440	7,322.879	7,389.242	7,325.876	—	—	—	1.00
2-type	−3,376.315	6,814.630	6,917.492	6,819.274	0.023	<0.001	0.900	0.64/0.36
3-type	−3,213.626	6,511.252	6,650.613	6,517.545	0.513	<0.001	0.946	0.62/0.06/0.32
4-type	−3,078.061	6,262.123	6,437.983	6,270.063	0.164	<0.001	0.941	0.06/0.54/0.31/0.08

**Figure 2:**
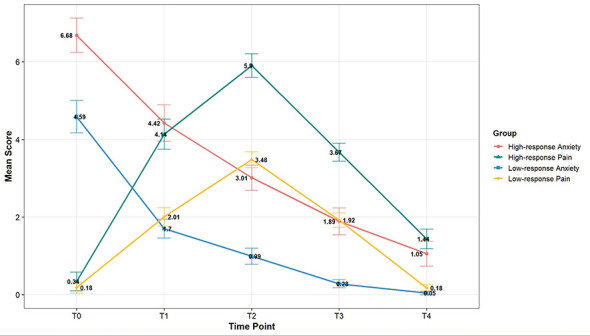
Perioperative anxiety and pain trajectories. The MDAS was administered at baseline to assess preoperative dental anxiety and was analyzed as a predictor variable. Perioperative anxiety trajectories were characterized using repeated VAS-A measurements at five time points: preoperative, intraoperatively, 6 h postoperatively, 24 h postoperatively, and 7 days postoperatively. Pain intensity was measured using the Visual Analog Scale for Pain (VAS-P) at the same perioperative time points.

Figure 2 demonstrates the trajectory of anxiety and pain scores at 5 time points for both potential categories. Patients in the Hyper-reactive category had significantly higher anxiety and pain scores than those in the Hypo-reactive category at all time points, and showed more dramatic fluctuations. In particular, pain scores increased rapidly during operation (T1), peaked at 6 h after operation (T2), declined gradually 24 h after operation (T3), and remained low at 7 days after operation (T4). The overall fluctuation of anxiety and pain in patients with the low response type was smaller and has faster recovery. At 7 days after operation (T4), the pain almost disappeared. Regardless of the high and low response types, the trends of anxiety and pain scores at time points were highly consistent, which shows that pain scores reaching a peak at 6 h after operation (T2) and then gradually decreasing as a trend of continuous relief.

### 3.2 Single factor analysis of perioperative period anxiety and pain profiles in dental implant patients

The differences between the two potential categories were statistically significant (*P* < 0.05) in terms of pain tolerance, implant acceptance type, presence of a caregiver, bone graft material, number of implants, MDAS, and oral health. The differences have statistically significant (*P* > 0.05) in terms of gender, age, smoking, education, spouse, occupation, and monthly income (Table 2).

**Table 2:** Univariate analysis of potential categories of perioperative anxiety and pain in dental implant patients.

Items	Group	*n*	Low-response group	High-response group	χ^2^/*t*	*P*
Gender	Male	92	65 (70.7%)	27 (29.3%)	3.021	0.477
	Female	112	66 (58.9%)	46 (41.1%)		
Age		204	48.14 ± 14.13	46.85 ± 12.41	1.189	0.516
Diploma	Bachelor degree or above	80	53 (66.3%)	27 (33.8%)	0.237	0.626
	Below undergraduate	124	78 (62.9%)	46 (37.1%)		
Spouse	Yes	176	113 (64.2%)	63 (35.8%)	0.000	0.993
	No	28	18 (64.3%)	10 (35.7%)		
Occupation	Employed	134	82 (61.2%)	52 (38.8%)	1.552	0.213
	Unemployed	70	49 (70.0%)	21 (30.0%)		
Monthly-income	<5,000	31	20 (64.5%)	11 (35.5%)	0.001	0.970
	>5,000	173	111 (64.2%)	62 (35.8%)		
Smoking	Yes	21	17 (81.0%)	4 (19.0%)	2.854	0.091
	No	183	114 (62.3%)	69 (37.7%)		
Drink	Yes	42	31 (73.8%)	11 (26.2%)	2.118	0.146
	No	162	100 (61.7%)	62 (38.3%)		
Oral disease	Yes	182	118 (64.8%)	64 (35.2%)	0.282	0.595
	No	22	13 (59.1%)	9 (40.9%)		
Unpleasant medical history	Yes	49	30 (61.2%)	19 (38.8%)	0.251	0.616
	No	155	101 (65.2%)	54 (34.8%)		
Pain tolerance	Excellent	40	34 (85.0%)	6 (15.0%)	16.023	**<0.001**
	Average	120	78 (65.0%)	42 (35.0%)		
	Poor	44	19 (43.2%)	25 (56.8%)		
Dental implant experience	Yes	15	13 (86.7%)	2 (13.3%)	3.551	0.059
	No	189	118 (62.4%)	71 (37.6%)		
Implant acceptance type	Proactive	160	109 (68.1%)	51 (31.9%)	4.934	**0.026**
	Passive	44	22 (50.0%)	22 (50.0%)		
Caregiver	Yes	91	59 (64.8%)	32 (35.2%)	0.027	0.868
	No	113	72 (63.7%)	41 (36.3%)		
Bone graft material	Yes	12	3 (25.0%)	9 (75.0%)	8.533	**0.009**
	No	192	128 (66.7%)	64 (33.3%)		
Implant time	Immediately	27	14 (51.9%)	13 (48.1%)	2.070	0.150
	Scheduled	177	117 (66.1%)	60 (33.9%)		
Teeth number		204	27.07 ± 2.62	26.37 ± 3.84	4.367	0.126
Number of dentures		204	1.77 ± 2.80	1.37 ± 2.30	3.021	0.335
Number of implants		204	1.52 ± 0.82	1.92 ± 1.12	2.080	**0.004**
MDAS		204	10.72 ± 2.32	13.55 ± 2.78	8.555	**<0.001**
Oral Health		204	24.60 ± 6.90	27.84 ± 8.68	4.000	**0.004**

### 3.3 Binary logistic regression analysis of perioperative anxiety and pain in profiles of dental implant outpatients

In order to further explore the predictors of the potential categories of perioperative anxiety and pain, the LPA classification results (low reactive as 0 and high reactive as 1) were used as the dependent variables, the variables that were statistically significant in the univariate analysis (*P* < 0.05) were selected to enter into binary logistic regression analysis. Prior to binary logistic regression analysis, multicollinearity diagnostics were performed. Tolerance values ranged from 0.636 to 0.943, and VIF values ranged from 1.060 to 1.573, indicating no evidence of substantial multicollinearity among the predictor variables. In addition, the Hosmer–Lemeshow goodness-of-fit test demonstrated adequate model fit (χ^2^ = 9.096, df = 8, *P* = 0.334). The results of the binary logistic regression analysis are shown in Table 3.

**Table 3:** Binary logistic regression analysis of perioperative anxiety and pain in dental implant patients across profiles.

Variable	Regression coefficient	Standard error	Wald	*P*	OR (95% CI)
Constant	−3.870	1.270	9.278	**0.002**	—
Smoking	−2.239	1.022	4.798	**0.028**	0.107(0.014–0.790)
Have caregiver	−1.492	0.566	6.956	**0.008**	0.225(0.074–0.682)
Average pain tolerance	−0.603	0.411	2.155	0.142	0.547(0.245–1.224)
Excellent pain tolerance	−0.783	0.655	1.428	0.232	0.457(0.127–1.651)
Receive implant passively	−0.521	0.395	1.736	0.188	0.594(0.274–1.289)
Using bone material	2.143	0.821	6.811	**0.009**	8.526(1.705–42.637)
Number of implant	0.458	0.174	6.903	**0.009**	1.581(1.183–2.224)
MDAS	0.242	0.072	11.190	**<0.001**	1.274(1.103–1.468)
Oral Health	0.013	0.023	0.294	0.588	1.013(0.968–1.060)

Smokers were significantly less likely to enter the high-response group (OR = 0.107, *P* = 0.028). The patients who unaccompanied by family members were significantly were easier to enter the high-response group (OR = 0.225, *P* = 0.008). The greater the number of implants, the more likely the patient was to enter the high-response group (OR = 1.581, *P* = 0.009). Using bone meal was the strongest predictor, such patients are more likely to enter the high response group (OR = 8.526, *P* = 0.009). The higher scores on the MDAS, the easier to enter the high-response group (OR = 1.274, *P* < 0.001). The probability of high response on the anxiety-pain response trajectory was elevated by approximately 27.4% for each one-point increase.

## 4 Discussion

Based on anxiety and pain assessments conducted at multiple key perioperative time points, this study adopted a person-centered latent profile analysis approach to explore potential heterogeneity in psychophysiological responses among patients undergoing dental implant surgery. The findings indicated that patients could be classified into two distinct perioperative anxiety–pain response profiles, namely a low-response profile and a high-response profile. Patients in the low-response profile consistently maintained relatively low levels of anxiety and pain throughout the perioperative period, whereas those in the high-response profile exhibited markedly elevated preoperative anxiety accompanied by persistently higher pain responses during intraoperative and postoperative stages. Notably, anxiety and pain trajectories within each profile demonstrated relatively stable and coherent response patterns across time, suggesting a close and dynamic coupling between anxiety and pain throughout the perioperative course. These findings indicate that dental implant patients do not respond to surgical stress in a uniform or homogeneous manner; instead, clinically meaningful differential response patterns exist, which provides an important empirical basis for implementing stratified and time-specific perioperative nursing management strategies.

Interestingly, pain intensity in both profiles reached its highest level at 6 h postoperatively and subsequently declined over time. This finding is broadly consistent with previous studies reporting an early postoperative pain peak following dental implant surgery (22), while differing from reports suggesting that peak pain may occur at later postoperative time points (23, 24). Such discrepancies may be attributable to differences in assessment schedules, surgical characteristics, and study populations across investigations. From a clinical perspective, the identification of an early postoperative pain peak highlights the first 6 h after surgery as a potentially critical period for timely pain management and supportive nursing interventions.

The observed differences between the two perioperative anxiety–pain response profiles in this study may be attributed to individual variations in cognitive appraisal, emotion regulation capacity, and neurophysiological sensitivity (35). Patients classified into the high-response profile were more likely to develop preoperative catastrophic expectations, characterized by negative anticipations regarding the surgical procedure and postoperative recovery accompanied by excessive worry (36). Under surgical stress, such maladaptive cognitions may amplify anxiety responses, thereby increasing pain sensitivity and lowering pain tolerance thresholds (37). This pattern is consistent with the fear–avoidance model (38), which posits that preoperative anxiety can enhance nociceptive processing through heightened sympathetic activation and emotional arousal, ultimately intensifying subjective pain experiences. In addition, interindividual differences in somatic signal processing and pain modulation may further contribute to this heterogeneity, as some patients may exhibit a tendency toward central sensitization, resulting in stronger and more persistent pain responses to comparable surgical stimuli (39).

In contrast, patients classified into the low-response profile maintained consistently low levels of anxiety and pain throughout the perioperative period, which may be attributable to more mature emotional regulation capacities and more adaptive cognitive coping strategies (40). This interpretation is supported by previous evidence. Ghobadi et al. (41) reported that persistent dental anxiety and pain experiences not only lead patients to delay or avoid dental care but may also indirectly increase surgical complexity thereby creating a self-reinforcing cycle in which anxiety and pain mutually exacerbate one another and ultimately exert long-term adverse effects on oral health outcomes (38). In addition, a study by Wang et al. focusing on older adults demonstrated that higher perioperative anxiety and pain levels were closely associated with sleep disturbances and delayed postoperative recovery, whereas the implementation of targeted psychological interventions resulted in significant reductions in anxiety and pain, accompanied by marked improvements in oral health status at postoperative day 7 (42). Collectively, these findings provide indirect support for the present results, suggesting that patients in the low-response profile may possess more favorable psychological adjustment characteristics, enabling a more stable and adaptive perioperative recovery trajectory.

Building on the identification of perioperative anxiety–pain response profiles, this study further employed binary logistic regression to examine predictors associated with different response patterns, thereby providing quantitative support for the observed heterogeneity. The findings indicated that preoperative dental anxiety emerged as one of the most robust predictors of classification into the high-response profile. Specifically, for each one-point increase in the Modified Dental Anxiety Scale (MDAS) score, the likelihood of belonging to the high-response group increased by approximately 27.4%. This result suggests that preoperative dental anxiety is not merely a transient emotional reaction but may exert a sustained amplifying effect on patients' pain perception and stress responses throughout the perioperative period. Such an interpretation is consistent with previous evidence linking elevated dental anxiety to pain catastrophizing and heightened threat anticipation, which in turn may intensify both psychological distress and subjective pain experiences during dental procedures (23, 43).

In addition to psychological factors, surgical-related characteristics may impose an additional physiological burden that contributes to heightened perioperative stress responses. In the present study, patients undergoing more complex implant procedures were more likely to exhibit a high anxiety–high pain response profile. In particular, individuals requiring concomitant bone grafting or a greater number of implants demonstrated a significantly increased likelihood of classification into the high-response group. These findings suggest that the extent of surgical trauma and the subsequent inflammatory response may intensify nociceptive input and pain sensitization, thereby amplifying psychophysiological stress reactions during the perioperative period. Collectively, this evidence further underscores the importance of incorporating surgical complexity into perioperative risk assessment frameworks to facilitate early identification of patients at elevated risk for adverse anxiety–pain responses (22, 43).

With respect to social factors, the presence of a caregiver during the perioperative period emerged as a significant protective factor. Patients attending appointments without accompaniment were more likely to be classified into the high-response profile, highlighting the important buffering role of social support in mitigating perioperative anxiety and pain responses. The presence of a caregiver may contribute to emotional reassurance, facilitate information exchange, and enhance patients' sense of safety, thereby promoting emotional stability and improving subjective appraisal of the surgical context (44). This finding provides empirical support for strengthening accompaniment and supportive interventions within clinical nursing practice. Notably, smokers in the present study were less likely to be classified into the high-response profile. However, this finding should be interpreted with caution because the number of smokers was relatively small and only a limited number were assigned to the high-response group, resulting in a wide confidence interval. Although previous studies have suggested that nicotine may transiently influence pain perception and stress responses (26), the underlying mechanisms remain uncertain and were beyond the scope of the present study. Importantly, substantial evidence has demonstrated that smoking adversely affects oral health and implant prognosis (45), and therefore this finding should not be interpreted as evidence supporting smoking as a strategy to reduce perioperative anxiety or pain. Further studies with larger samples are needed to verify this association. Overall, the regression findings reinforce the multidimensional determinants of perioperative anxiety–pain responses, suggesting that high-response patterns are not driven by a single factor but rather arise from the combined influence of psychological vulnerability, surgical complexity, and social support. These findings underscore the importance of comprehensive early risk assessment to identify high-risk patients and provide a foundation for the implementation of stratified and precision-oriented perioperative nursing management strategies.

This study highlights the importance of early identification and stratified management of perioperative anxiety–pain responses in patients undergoing dental implant surgery. Through multi–time-point assessments and response profile classification, nurses are better positioned to implement person-centered and precision-oriented intervention strategies. In clinical practice, particular attention should be directed toward patients classified into the high-response profile, with supportive measures tailored according to their preoperative anxiety levels, surgical complexity, and available social support. Such measures may include enhanced health education, the use of non-pharmacological interventions (e.g., music therapy), and the facilitation of family accompaniment when feasible. In contrast, patients in the low-response profile may primarily require the maintenance of routine education and psychological support to reinforce adaptive coping strategies. Effective perioperative anxiety–pain management relies on a multidisciplinary, nurse-led care model in which nurses play a central role in risk identification, continuous monitoring, and intervention delivery. The implementation of stratified nursing strategies based on response profiles may help alleviate patients' negative experiences, improve the quality of implant-related nursing care, and ultimately enhance patient satisfaction with treatment.

Several limitations of this study should be acknowledged. First, this study employed a single-center convenience sampling strategy with a relatively limited sample size, which may have introduced selection bias and reduced the representativeness of the study population. Moreover, no *a priori* sample size calculation was performed. Although the overall sample size was considered adequate for the present analyses, several subgroups were relatively small, particularly patients receiving bone graft materials and smokers. Therefore, the corresponding findings should be interpreted with caution. Second, the potential influence of unmeasured confounding factors cannot be fully ruled out, including individual personality traits, variations in surgeons' technical proficiency, and the precision of intraoperative anesthetic administration, all of which were difficult to comprehensively control within the present study design. In addition, intraoperative anxiety and pain were assessed immediately after surgery based on patients' retrospective reports rather than real-time measurements. Although this approach minimized interference with the surgical procedure, recall bias cannot be completely excluded. Finally, as this investigation was conducted at a single center, the generalizability of the findings may be constrained. Future multicenter studies with larger and more diverse samples are warranted to further validate the present findings and to provide more robust evidence to support the development of comfort-oriented and precision-based perioperative nursing care in dental implant practice.

## 5 Conclusion

This study identified two distinct perioperative anxiety–pain response profiles among patients undergoing dental implant surgery: a low-response profile and a high-response profile. Perioperative anxiety and pain exhibited heterogeneous dynamic patterns, with the high-response subgroup demonstrating persistently elevated symptom levels, a slower recovery trajectory, and a peak psychological–physiological response at 6 h postoperatively. This subgroup represents a clinically important population requiring closer monitoring and enhanced supportive care. Further analyses demonstrated that binary logistic regression is a useful approach for examining factors associated with these response patterns. Preoperative anxiety levels, surgical complexity, and social support emerged as key predictors of profile membership, underscoring their relevance in perioperative assessment and clinical planning. Collectively, these findings highlight the necessity of integrating targeted, profile-informed interventions into nursing practice. Early identification of individuals in the high-response group, followed by timely and tailored management, may help alleviate perioperative distress, facilitate recovery, and mitigate the broader impact of heightened anxiety–pain responsiveness among patients receiving dental implants.

## Data Availability

The raw data supporting the conclusions of this article will be made available by the authors, without undue reservation.
